# Exon capture and bulk segregant analysis: rapid discovery of causative mutations using high-throughput sequencing

**DOI:** 10.1186/1471-2164-13-649

**Published:** 2012-11-21

**Authors:** Florencia del Viso, Dipankan Bhattacharya, Yong Kong, Michael J Gilchrist, Mustafa K Khokha

**Affiliations:** 1 Department of Pediatrics and Genetics, Yale University School of Medicine, 333 Cedar Street, New Haven, CT, 06520, USA; 2 Department of Molecular Biophysics and Biochemistry and W. M. Keck Foundation Biotechnology Resource Laboratory, Yale University School of Medicine, 333 Cedar Street, New Haven, CT, 06520, USA; 3 Division of Systems Biology, MRC-National Institute for Medical Research, Mill Hill, London, NW7 1AA, UK

**Keywords:** Exon capture sequencing, Forward genetics, *Xenopus tropicalis*, Bulk segregant analysis, Cilia, Kidney development, SNP discovery, Genome assembly, ccdc40, pax8

## Abstract

**Background:**

Exome sequencing has transformed human genetic analysis and may do the same for other vertebrate model systems. However, a major challenge is sifting through the large number of sequence variants to identify the causative mutation for a given phenotype. In models like *Xenopus tropicalis*, an incomplete and occasionally incorrect genome assembly compounds this problem. To facilitate cloning of *X. tropicalis* mutants identified in forward genetic screens, we sought to combine bulk segregant analysis and exome sequencing into a single step.

**Results:**

Here we report the first use of exon capture sequencing to identify mutations in a non-mammalian, vertebrate model. We demonstrate that bulk segregant analysis coupled with exon capture sequencing is not only able to identify causative mutations but can also generate linkage information, facilitate the assembly of scaffolds, identify misassembles, and discover thousands of SNPs for fine mapping.

**Conclusion:**

Exon capture sequencing and bulk segregant analysis is a rapid, inexpensive method to clone mutants identified in forward genetic screens. With sufficient meioses, this method can be generalized to any model system with a genome assembly, polished or unpolished, and in the latter case, it also provides many critical genomic resources.

## Background

High throughput sequencing (HTS) has revolutionized our ability to analyze genomes for mutations that cause disease phenotypes. Whole genome sequencing (WGS) can identify causative mutations in smaller invertebrate genomes such as *D. melanogaster*[1] and *C. elegans*[2] and also larger vertebrate genomes such as mouse and zebrafish [3-5]. However, most disease causing mutations are in exons, and WGS of vertebrate genomes remains relatively expensive compared to exome sequencing. Therefore, for disease mutation discovery, exome sequencing is high-yield at a minimum cost.

To perform exome sequencing, exons are captured using arrays [6-8] or in solution [9] with specifically designed probes. Since the exome represents <5% of genomes, a fraction of an Illumina HiSeq lane can generate sufficient coverage depth for confident variation detection. Much more sequencing is necessary for WGS. Exome sequencing has successfully led to discovery of mutations in polished genomes such as human and mouse [10-17].

However, exon capture HTS is limited by the quality of the genome assembly and annotation. For example, in *X. tropicalis*, a frog genetic model system, many of the exons have been identified, yet at least 5% of the genome, including exonic sequence, is in gaps in the latest genome assembly (v7.1) [18]. These difficulties also affect the analysis of zebrafish and other emerging model systems. In addition, even in polished genomes, a major challenge in the analysis of exomes is to identify a causative mutation amongst the large array of natural sequence variations present in the genome. In forward genetic screens, heavily mutagenized genomes have additional changes, further complicating discovery of causative mutations.

Using *X. tropicalis*, we aimed to circumvent these difficulties by combining exon capture HTS with bulk segregant analysis (BSA). In BSA, carriers of a recessive phenotype are crossed to produce wildtype and mutant progeny. These progeny are sorted based on their phenotype into two pools (or bulks), mutant and wildtype. Genetic markers (e.g. heterozygous SNPs, microsatellites, or RFLPs) are then assayed on the bulk DNA. If the bulks contain a sufficient number of embryos (i.e. meioses), then unlinked genetic markers will be detected as heterozygous in both pools. On the other hand, genetic markers linked to the causative mutation will appear homozygous in mutant pools but will remain heterozygous in wildtype pools. This loss of heterozygosity (LOH) in mutant pools indicates linkage and can be used to identify candidate loci. By combining data from BSA and exon capture sequencing, we can sift through the large array of sequence variations to identify a region containing the mutant gene. In polished genomes such as mouse, exon capture/BSA can be adapted to narrow candidate mutations in a single step at much lower costs than WGS.

*Xenopus* is an extraordinarily valuable system for biomedical research, but forward genetic analysis only became effective with the introduction of the diploid *X. tropicalis* as a model [19-22]. Employing forward genetic screens, numerous mutants have been identified [23-25], but discovering the underlying causative mutations is difficult due to the relative paucity of genetic markers and limitations of the long-range genome assembly. Despite these difficulties, some causative mutations have been characterized [26-28]. To facilitate the discovery of mutations in *X. tropicalis*, we sought to incorporate meiotic data into our exon capture HTS by adding BSA into the sequencing step. We show that exon capture HTS with BSA facilitates local genome assembly, genetic marker identification, and causative mutation discovery. Using this method, we were able to identify candidate genes for two *X. tropicalis* mutants in just a few weeks. We then verified the identity of the mutated gene using either mRNA overexpression to rescue the phenotype, or morpholino knockdown to phenocopy. In one case, gaps in the genome and local misassembly would have made mutation identification extremely difficult without exon capture/BSA. This method produces genomic resources inexpensively and can quickly identify candidate mutations amongst many sequence variations.

## Results

**Figure 1:**
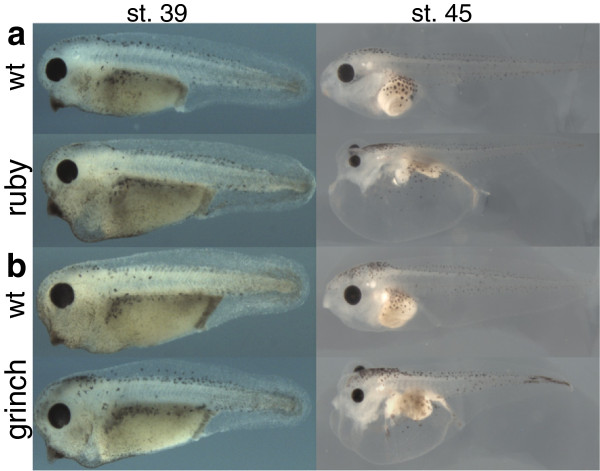
***ruby *and *grinch *mutant phenotypes. ***ruby* (**a**) and *grinch* (**b**) mutant phenotypes start with pericardial edema at stage (st.) 39. At st. 45, the edema worsens and is lethal. Wildtype (WT) embryos from the same cross are shown for comparison. All embryos are lateral views with dorsal to the top and anterior to the left.

In a forward genetic screen in *X. tropicalis,* we identified two mutants, *ruby* and *grinch* that follow simple Mendelian inheritance, show similar phenotypes, but fall into different complementation groups (Figure 1). Both mutants appear wildtype until stage 38–39 when edema starts to appear around the heart, steadily worsens, and finally causes death by stage 46–48 (Figure 1a,b). The etiology of the edema is unclear and could be due to cardiac, lymphatic or renal defects [29-34]. To determine the causative genes, we developed a HTS approach combining exon capture with BSA. First, we developed an exon capture array containing coding exons identified in the published *X. tropicalis* v4.1 genome [35]. Because approximately 5-10% of the genome is missing from this assembly [18,35], we also identified coding exons from available EST clusters and full-length mRNA sequences to augment the array.

**Figure 2:**
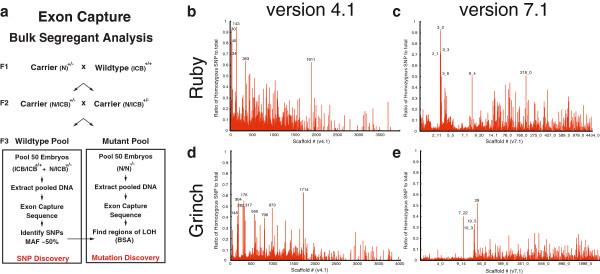
**BSA and Exon Capture HTS.** (**a**) Schematic of Exon Capture HTS with BSA. Two pools of embryos are sequenced, one mutant and one WT from a mapcross. The WT pool is sequenced and SNPs identified based on allele counts where the major allele fraction (MAF) is approximately 50%. These positions are examined in the mutant pools for LOH. (**b**,**d**) Graph of homozygosity ratio to scaffold number using genome v4.1 for *ruby* (**b**) and *grinch* (**d**). (**c**,**e**) same as **b**,**d** but version 7.1 of the genome. In (**b**-**e**), scaffolds or scaffold intervals with <20 SNPs have been excluded. The peaks with the highest homozygosity ratios are labeled.

We then captured and sequenced coding exons and flanking intronic sequence from DNA of pooled embryos, in order to exploit BSA. Hybrid carriers from our mutagenized strain (N) and mapcross strain (ICB or PopA) were crossed to generate mutant and wildtype embryos. Two pools of these embryos were collected: one pool had only phenotypically mutant (MUT) embryos and the other had wildtype (WT) embryos (Figure 2a). We performed exon capture using our custom arrays and, using the Illumina platform, sequenced each pool individually (see Methods). We first analyzed the WT pool for heterozygosity, which identified SNPs in these populations. In mutant pools, we examined these same genomic positions for LOH suggesting linkage to the causative mutation (Figure 2a and Methods). Our analysis identified over 30,000 putative SNPs that were common across all mutant and WT pools, with a subset of SNPs becoming homozygous in the mutant pools.

For the v4.1 genome assembly, we calculated a homozygosity ratio by dividing the number of homozygous SNPs to heterozygous SNPs per scaffold. We focused on scaffolds that had at least 20 SNPs per scaffold to avoid bias from falsely called SNPs (Figure 2b-e). For *grinch*, these excluded scaffolds (with <20 SNPs) represent less than 0.7% of the genome, and for *ruby*, this represents less than 0.9% of the genome. Although this introduces the risk of missing smaller scaffolds that are linked to the mutation, this filter helped clarify and prioritize our analysis. When we had completed this analysis, the v7.1 genome assembly became available so we repeated the analysis on this assembly. The v7.1 genome assembly employed a preliminary *X. tropicalis* meiotic map as well as synteny to other genomes to create chromosome-sized super-scaffolds [18]. In this case, for all scaffolds larger than 0.5 Mb, we divided the scaffold into 0.5 Mb intervals and calculated the homozygosity ratio for these smaller intervals, again excluding intervals that had less than 20 SNPs from further analysis. This analysis identified scaffolds or genomic intervals with high homozygosity ratios indicating linkage to the causative mutation in *ruby* and *grinch* mutants (Figure 2b-e).

### Cloning *ruby* phenotype

**Figure 3:**
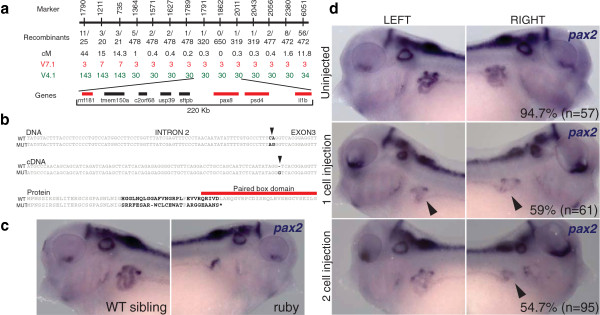
**Mapping of *ruby *mutation.** (**a**) Mapping interval derived from exon capture and BSA. The number of recombinants for each RFLP marker, the genetic distance and the location of the marker in v4.1 and v7.1 genome assemblies are shown. The 220 kb interval contains 7 genes: *tmem150a*, *c2orf68*, *usp39*, *sftpb*, *pax8*, *psd4* and a portion of *il1b*. Genes in red have mutations causing amino acid or splice site changes based on the exon capture data. (**b**) Genomic, cDNA and protein sequences of WT and mutant *pax8*. The mutation of two contiguous nucleotides (nt) in intron 2 (arrowhead) shifts the acceptor splice site 1 nt, causing the inclusion of an extra G in the transcript (cDNA) (arrowhead). The protein sequence shows a change in frame and shortly after a premature STOP codon (*) abrogating the entire paired box domain. (**c**) *Pax2* expression in wt and mutant *ruby* embryos by Whole Mount *in situ* hybridization showing clear impairment of pronephric development (arrowhead). (**d**) *Pax8* morpholino phenocopies *ruby* phenotype. *Pax8* morpholino was injected at one or two-cell stage (single cell injected). Embryos were fixed at st. 36–37 and *pax2* expression was used to assess pronephric development. The embryo shown for two-cell stage injection was injected on the right side (based on fluorescent dye tracer [not shown]). Arrowheads indicate abnormal pronephros. Embryos in (**c**,**d**) are lateral views with dorsal to the top and anterior to the left (in left columns) or right (in right columns).

Prior to this analysis, we had no previous mapping information for *ruby*. Exon capture/BSA from mutant *ruby* embryos found six v4.1 scaffolds with high LOH signal (Figure 2b) and a striking signal in scaffold 3 in the v7.1 genome (Figure 2c). Using our newly identified SNPs, we sought to both validate our SNP discovery and narrow down the interval carrying the causative mutation. For fine mapping purposes, we focused on SNPs that led to Restriction Fragment Length Polymorphisms (RFLPs) and found that 46/51 SNPs (90%) tested had the expected polymorphism. We then showed that three of the scaffolds from v4.1 (30, 34, 143) were tightly linked to the *ruby* locus, thus effectively assembling the scaffolds at this locus (Figure 3a). Analyzing the v7.1 assembly, we confirmed that scaffold 3 contains the *ruby* locus and identified a misassembly of a portion of scaffold 7 (Figure 3a). Although we did not test markers on scaffolds 40 and 363 (v4.1) (Figure 2b), in the v7.1 genome, both scaffolds are incorporated into scaffold 3 and therefore are likely linked to the mutation. Scaffolds 1911 (v4.1) and 218 (v7.1) may be linked to the *ruby* locus, but were not tested since we mapped *ruby* to a 220 kb or 0.3 cM interval on scaffold 3 (v7.1) using 650 mutant embryos.

This interval contains only 7 genes (Figure 3a), and analysis of exon capture sequence revealed that three of these genes, *il1b*, *psd4*, and *pax8,* had sequence variants leading to amino acid changes or splice site changes. *Il1b* had one amino acid change but was outside the mapped interval (markers 1791–2011). *psd4* had 3 amino acid changes that were all conservative. More importantly, the expression pattern of *psd4* did not support a role in *ruby*, as there appears to be little expression prior to the onset of the phenotype (Additional file 1: Figure S1).

*Pax8* had a mutation in the acceptor splice site of intron 2, causing a mis-splice and frameshift, eliminating the entire paired-box domain (Figure 3b). We confirmed this splice site mutation by RT-PCR and Sanger sequencing (Figure 3b cDNA). *Pax8* has previously been shown to be a principal and early regulator of pronephric development in *Xenopus*[36]. Furthermore, mutant embryos had a reduced expression of *pax8* (Additional file 1: Figure S2) and defects in pronephric development as shown by *pax2* (Figure 3c) and several other pronephric markers [37] (Additional file 1: Figure S2). This is consistent with the edema phenotype, especially because the onset of the edema is shortly after the pronephros becomes functional [38].

To demonstrate that *pax8* is the causative gene for the *ruby* phenotype, we used an antisense translation blocking morpholino (MO) to knockdown *pax8*. Injection of *pax8* MO at the one cell stage caused a clear impairment of pronephric development in 59% of the embryos (Figure 3d) phenocopying *ruby* mutants. In *Xenopus*, we can target either the left or right pronephros by injecting one cell at the two-cell stage. When targeted to one side, *pax8* knockdown causes impairment in pronephric development in 54.7% of the embryos on the injected side (Figure 3d and Additional file 1: Figure S3). Taken together, these results indicate that the phenotype in *ruby* mutants is caused by a *pax8* mutation that leads to a truncated protein and disrupts pronephric patterning at early stages of development.

### Cloning *grinch* phenotype

**Figure 4:**
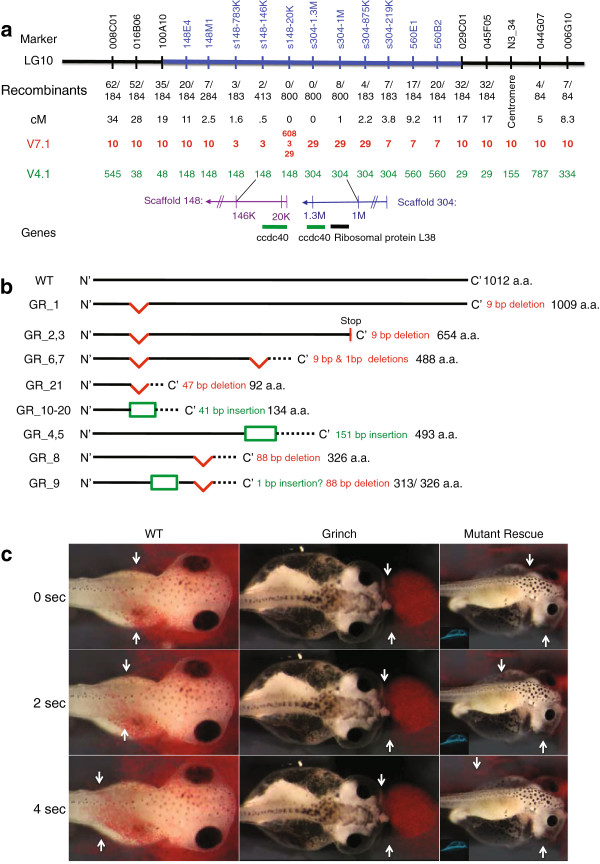
**Mapping the *grinch *mutation.** (**a**) Local genome assembly of the *grinch* locus using exon capture/BSA. The blue region marks the 10 cM gap in the meiotic map. The number of recombinants for each marker, calculated genetic distance and the location of the markers in v7.1 and v4.1 genome assemblies are shown. The 400 kb interval contains 2 genes: *ccdc40* on the ends of scaffold 148 and 304 (v4.1) and *ribosomal protein L38*. (**b**) Schematic diagram of the protein sequences of the 21 mutant clones. Red regions indicate deletions, green box indicates insertions, and dashed lines indicate frameshifts. Number of bp inserted or deleted and translated amino acid (a.a.) length for each clone is indicated. See Additional file 1: Figure S5 for complete mutant protein and cDNA sequence. (**c**) Cilia-driven epidermal flow study using red microbeads. Arrows follow a particular bead’s trajectory through time. In left column, WT embryo shows equal flow on both the left and right sides. In middle column, *grinch* mutants show no flow on either side. In right column, mutants injected with WT *ccdc40* mRNA and GFP tracer (insert) show rescue of flow on injected side. See Additional files 1– 4. Embryos in (**c**) are dorsal views with anterior to the right.

Similar to *ruby*, *grinch* mutants also develop significant ventral edema, but unlike *ruby*, *grinch* mutants have a ciliary defect that can be detected by reduced cilia-driven flow on the epidermis of mutants (Figure 4c, Additional file 2 and Additional file 3). Scanning Electron Microscopy (SEM) also demonstrates fewer and abnormal cilia in multi-ciliated epidermal cells (Additional file 1: Figure S4).

However, mapping the *grinch* mutation has been difficult from the start. *grinch* is a background mutation in *X. tropicalis*, identified in numerous unrelated animals from different labs. They all cause the same edema phenotype and based on complementation testing are at the same loci. This and the fact that the phenotype follows simple Mendelian inheritance suggest a mutation at a single gene locus, with the possibility of more than one mutant allele. Furthermore, preliminary mapping linked the *grinch* mutation to a 10 cM gap in the meiotic map on chromosome 10 (Figure 4a) [39]. Scaffolds spanning the interval were unknown in the v4.1 assembly, making traditional positional cloning difficult.

We employed exon capture with BSA to identify linked scaffolds. We identified nine scaffolds in v4.1 that appeared linked to this locus (Figure 2d). Aligning our exome sequences with v7.1 scaffolds, we demonstrated linkage to chromosome 10, consistent with our previous mapping. We also found linkage to scaffold 29 and a portion of chromosome 7, which indicates a misassembly (Figure 2e). Of note, the homozygosity signal in the *grinch* mutant pools was lower than for *ruby* (see below).

We next sought to validate these scaffolds as linked to the *grinch* locus by meiotic mapping on a panel of mutant embryos. This again highlights the power of exon capture/BSA sequencing. With thousands of newly identified SNPs, some of which were RFLPs, we used meiotic mapping to create our own local assembly and fill the gap containing the *grinch* locus (Figure 4a). We successfully amplified 14/17 SNPs/RFLPs and 12 of them (86%) had the expected polymorphism. Fine mapping revealed that the interval containing the mutation is at the ends of scaffold 148 and 304 (v4.1). Both scaffolds share the *ccdc40* gene suggesting that either these two scaffolds are overlapping or are adjacent with a local gene duplication. In the v7.1 genome, *ccdc40* is triplicated on three different scaffolds, chromosome 3, scaffold 29, and scaffold 608 (Figure 4a). In addition, one other gene, *ribosomal protein L38*, fell within our genetic interval. The other v4.1 scaffolds (176, 282, 317, 796, 973 and 1714) indicated by our exon capture/BSA analysis (Figure 2d) are all assembled into scaffold 10 in v7.1. Therefore, although not tested by fine mapping, they are all likely linked to the mutation.

*ccdc40* in *X. tropicalis* is an 18 exon, 3033 bp transcript. Mutations in CCDC40 were recently found in patients with Primary Ciliary Dyskinesia [40,41]. SEM and Transmission Electron Microscopy (TEM) images of *grinch* cilia show similar microtubule displacement as well as shorter and fewer cilia consistent with the human disease [40] (Additional file 1: Figure S4). To assess alterations in the transcript, we analyzed pools of mutant or wt embryos and performed RT-PCR using gene specific primers within the 5’ and 3’ UTR regions. We then cloned and sequenced the PCR products. Overall, 20/21 clones from mutants have open reading frames that lead to truncated proteins. The remaining clone has a deletion of three consecutive amino acids (Figure 4b and Additional file 1: Figure S5). In the WT pool, which includes WT embryos as well as heterozygote carriers, 17/19 of the clones are WT. Of the two others, one has a premature stop codon and the other an internal deletion, which are both found in the mutant transcripts.

It is interesting to note that we found heterozygous SNPs in the mutant transcripts, suggesting two different mutant alleles (Additional file 1: Figure S5). This was also confirmed in exon capture sequence and may explain the relatively low homozygosity ratios seen in Figure 2d,e (see Discussion).

Within the 21 mutant transcripts cloned (Figure 4b), we found several different mutations, most of which show features of aberrant splicing leading to frameshifts and premature stop codons (Additional file 1: Figure S5). 17/21 of the clones (1–3, 6,7, 10–21) suggest a splicing defect between exons 2 and 3, but neither genomic sequencing nor the exon capture data elucidated any obvious mutations in the splice acceptor/donor sites flanking or within intron 2. *grinch* clones 4 and 5 (also 10, 11, 13, 14, 16–19) retain intron 9 although again, we detect no mutations in the genomic or exon capture sequences (Additional file 1: Figure S5b). Clones 8 and 9 have a deletion of 88 bp within exon 9, whether this is due to aberrant splicing is uncertain. We consider the 1 bp insertion in clone 9 to be a PCR and sequencing artifact since it occurs after a string of “A” nucleotides and has not been detected in any other clones (Additional file 1: Figure 5b). Nevertheless all the mutant transcripts for *ccdc40* are abnormal compared to wildtype.

To confirm *ccdc40*, we attempted to rescue the cilia-driven epidermal fluid flow using mRNA microinjection. We injected WT *ccdc40* mRNA along with a GFP tracer into one cell at the two-cell stage embryos. We assayed for cilia-driven flow over the surface of the embryo using colored beads and then genotyped the embryos. *ccdc40* mRNA injection rescued the severe reduction in epidermal flow in *grinch* mutants only on the injected side in 51/55 genotyped mutants (Figure 4c and Additional file 4 and Additional file 5).

Unfortunately, identifying the exact nucleotides mutated in *grinch* has not been possible despite exon capture or genomic sequencing. It is not clear if the mutant variants are due to more than one allele or a single allele that causes multiple abnormalities in splicing. However, taken together, mapping, transcript sequencing, imaging of cilia, and rescue are all consistent with *ccdc40* mutation(s) causing the *grinch* phenotype. Given the complexity of the locus, exon capture/BSA was essential to identify this gene.

## Discussion

Exon capture sequencing has transformed genetic analysis in humans and has demonstrated utility in mice. However, to date, exon capture HTS has been scarcely reported in other genetic models [42,43] despite the significant need to facilitate cloning of mutants identified in forward genetic screens. Very recently, WGS with BSA has identified causative mutations in both zebrafish and mouse validating HTS and BSA as an effective method [4,5]. For smaller genomes, such as yeast, worms, flies or *Arabidopsis* WGS is probably cost efficient, therefore there is no need to pre-capture the exome. However, in model systems with large genomes, currently the cost of WGS remains relatively expensive compared to exome sequencing, especially for routine use in the large numbers of mutants. In addition, to maximize the power of BSA, depth of sequencing is useful to accurately identify SNPs especially given the challenges of unambiguously mapping short read sequences and sequence errors. In our mapping intervals, we validated ~90% of the SNPs identified by exon capture/BSA. Finally, exome sequencing is high-yield since most disease mutations are in exons [44].

Despite the advantages of exome sequencing, a number of barriers exist including unpolished genome assemblies, partial genome annotation, incomplete or poor characterization of variations in strains, and incomplete inbreeding of strains. As a consequence, deciphering causative mutations from the sea of variants within an exome can be a significant challenge. There are also limits to exon capture sequence regardless of genome quality or model organism. Exon capture sequencing only identifies exons and nearby flanking sequence so causative mutations deep in introns, promoters, or enhancers can be missed. Also, insertions, deletions, and splice variants may be missed by exon capture sequence due to limitations in alignment software and short read sequencing. In each of these cases, linkage information can ameliorate these problems.

The identification of the causative mutation in *ruby* was rapid and the significant advantage of exon capture/BSA sequencing was that many variants could be eliminated, thousands of genetic markers identified, and a relatively narrow interval analyzed for a causative mutation within the exon capture sequence. No preliminary mapping is necessary and the meiotic interval can be narrowed based on available meioses.

In the case of *grinch*, positional cloning was difficult. The linked interval was not present in the meiotic map and misassembled in both versions of the genome. Exon capture/BSA identified a large number of markers and scaffolds within the gap that allowed us to assemble the *grinch* locus.

However, we observed some heterozygosity at the *grinch* locus, suggesting more than one mutant allele. An additional allele could have been introduced since *grinch* was identified in numerous unrelated animals from different labs. They all cause the same edema phenotype and based on complementation testing are at the same loci. Of note, the homozygosity ratios are lower for *grinch* compared to *ruby*. The presence of another mutant allele could explain the lower homozygosity signals since each allele may be associated with different SNPs and therefore appear heterozygous. Alternatively, the mutant transcripts could be due to a single allele causing multiple abnormalities in splicing.

Regardless, all mutant transcripts had either an internal deletion or a frameshift leading to a premature stop codon. Alignment with other species shows that the C-terminus of the protein is most conserved, and we hypothesize that these truncated proteins will be non-functional. Furthermore, microinjection of the WT *ccdc40* mRNA into mutant embryos was sufficient to rescue cilia-driven epidermal flow. Thus, despite the complexity at the *grinch* locus, which would have made traditional positional cloning very difficult, using exon capture/BSA, we were able to identify *ccdc40* mutations as causative of the *grinch* phenotype.

Additionally, because exon capture/BSA is independent of the genome assembly, it can detect misassemblies. In fact, WGS/BSA experiments have identified unexpected linkage across disparate parts of the genome [4,5]. The cause was not further investigated in these studies so genome misassembly or inaccurate SNP calling remain a possibility. In our study, we found evidence of misassembly in our exon capture/BSA data for both *ruby* and *grinch* and validated these misassemblies by meiotic mapping. Once identified by exon capture/BSA, we can use meiotic mapping to reassemble the genome and continue fine mapping.

## Conclusions

Based on our study, we propose the following method to rapidly clone causative mutations in model systems with large genomes, such as *Xenopus*, zebrafish, and mice. First, an exon capture array needs to be generated using exons from a draft genome assembly and/or transcripts from ESTs, mRNA full-length sequencing, or transcriptome studies using next generation sequencers. Second, exon capture sequencing of a wildtype pool is necessary if SNPs are not well characterized in the model system. As more individuals in a strain are sequenced, wildtype sequencing becomes less essential. Third, exon capture sequencing in a mutant pool is done to identify regions of LOH. Exon capture sequence can then be analyzed for deleterious mutations in those regions. However, in more challenging cases where the LOH interval is large or complex, we recommend validating linkage by additional fine mapping to narrow the meiotic interval further or reassemble the locus. Lastly, once a candidate gene is identified, an independent validation is necessary such as a second allele, MO phenocopy, or rescue.

For model systems with large and unpolished genomes, exon capture HTS with BSA can be critical for identifying causative mutations because so many genomic resources can be obtained inexpensively. The cost of HTS is continuously decreasing and by using multiplexing techniques like barcoding, a single Illumina sequencing lane can generate sufficient sequence at considerable depth for multiple mutants. This represents a considerable cost savings compared to WGS. Furthermore, exon capture with BSA identifies a host of SNPs, facilitates local genome assembly, and discovers candidate mutations. This can be done very quickly (weeks) compared to the laborious methods such as chromosome walking across gaps.

For model systems with polished genomes, bulk segregant analysis and HTS can rapidly identify candidate genes within a narrow locus all in a single sequencing step. Finally, developing methods to rapidly and inexpensively clone mutants strongly encourages additional forward genetic screens to discover phenotypes and gene mutations that will yield fascinating new insights.

## Methods

### Frog husbandry

*X. tropicalis* were housed and cared for in our aquatics facility according to established protocols that were approved by our local IACUC.

### Mutant identification

We identified both *grinch* and *ruby* in a forward genetic screen using gamma-ray irradiation as a mutagen. We immediately recognized that *grinch* was in the background of our N strain since multiple, even unmutagenized animals generated the same *grinch* phenotype [24]. Other labs also identified a *grinch* phenotype (Lyle Zimmerman, Robert Grainger, personal communication), which based on complementation testing were mutations at the same loci and at the time thought to be identical alleles.

*ruby* was identified from a single mutagenized founder in the N strain. Although the *ruby* phenotype was similar to *grinch*, complementation testing revealed that they were at different loci. *ruby* and *grinch* animals were crossed to our mapcross strains, ICB or PopA respectively for analysis.

### DNA extraction for exon capture

Either 50 mutant or wildtype tadpoles at stage 42 were collected in 15 ml of lysis buffer (20 mM Tris pH 7.5-8.0, 100 mM NaCl, 20 mM EDTA, 1% SDS) supplemented with 200 mcg/ml proteinase K. Tube was rotated end over end at 55°C overnight. A phenol extraction was followed by a phenol:chloroform extraction, and then the DNA was precipitated with isopropanol, washed with 70% ethanol, and resuspended in TE buffer. DNA was processed for exon capture sequencing according to established protocols [10].

### Exon capture and sequencing analysis

We generated an exon capture array by identifying all exons in gene models present in the *X. tropicalis* v4.1 genome [35]. We also analyzed full-length mRNA sequences and EST clusters to see if they mapped to the genome. Unmapped sequences (due to gaps in the genome) were also added to the exon capture array. Roche-Nimblegen technical support generated probes against these targets (gene models and unmapped transcripts) using their in-house proprietary software and then manufactured the arrays.

For *ruby*, we generated 1 lane of 100 bp paired-end Illumina GAIIx sequencing data for each wildtype (WT) and mutant (MUT) pool generating 36.1 M pairs of reads for WT and 34.0 M for MUT. For *grinch*, we generated 2 lanes of 100 bp paired-end Illumina GAIIx data for each pool with 68.2 M pairs of reads for WT and 67.2 M for MUT. The Illumina reads were first trimmed based on their quality scores using Btrim [45]. We used a cutoff of 25 for average quality scores within a moving window of 5 bp. A minimum acceptable read length is 25 bp. Other parameters for Btrim were set at defaults. After the quality trimming, 96% of reads were kept for *ruby* WT and 89% for MUT. For *grinch*, the trimming passing rates were 91% and 93% for WT and MUT respectively. The trimmed reads were mapped to the reference genomes (v4.1 [accession number AAMC00000000] and v7.1 [accession number AAMC02000000] available at http://www.xenbase.org) using Burrows-Wheeler Aligner with default parameters [46]. The mapping results were converted into bam format first and then pileup format using samtools [47].

Using the two pileup files from WT and MUT as inputs, we created a script to analyze the alleles and their frequencies along each position of the genome with mapped reads. At each position, a minimum of 10 mapped reads in both WT and MUT pools were required for analysis. We identified a putative SNP if (i) the major allele frequency (MAF) in reads of WT is in the range of 50% and 75%; or (ii) WT major allele differs from that of MUT.

For all putative SNPs, we counted the number of homozygous SNPs (nh) in the MUT pools where MAF ≥ 90%. Then the homozygosity ratio (r) of homozygous SNPs in a region is calculated as r = nh/nt, where nt is the total number of SNPs in the same region. For reference genome v4.1, we used each scaffold as a region. For reference genome v7.1, since the scaffolds are much longer, non-overlap sliding windows with a size of 0.5 Mb was used for a region. Detailed protocols and scripts for mapping sequence data and calling SNPs is available at our website (tropicalis.yale.edu/geneticResources/geneticResources_main.html), and SNP data is available per request.

### Mapping

RFLP markers were designed in regions with LOH. All the SNPs in these regions were evaluated with WAT-CUT (http://watcut.uwaterloo.ca/watcut/watcut/template.php) to generate primers flanking SNPs that create or abolish a restriction site (RFLP markers). Primers were designed to amplify at least 100–250 bp flanking a SNP so we could resolve genotypes on a simple 4% agarose gel. A list of all the primers used is described in Additional file 1: Table S1. PCR with each pair of primers was performed in 96-well plates using genomic DNA from a large panel of individual mutant embryos and WT embryos as controls. PCR conditions were as follows: 94°C for 2 min, followed by 39 cycles at 94°C for 10 s, 58°C for 30 s and 72°C for 30 s. Final extension was at 72°C for 5 min. PCR products were digested with the corresponding enzyme either 4 h or overnight at 37 º C and run in 4% 50:50 regular agarose (American Bioanalytical): NuSeive agarose (LONZA) gels.

Preliminary mapping of *grinch* was done using primers designed around Simple Sequence Length Polymorphisms (SSLPs) as identified by the meiotic map. PCR conditions were the same as above. Primers 148E4, 148 M1, s304-875 K, s304-219 K, 560E1, and 560B2 were designed around SSLPs with a florescent 5’-m13 tail on the Forward primer [48]. PCR was done in 2 steps. Step 1: Using 5’-m13-F primer and R-primer; PCR conditions were 94°C for 2 min, followed by 34 cycles at 94°C for 10 s, 58°C for 30 s and 72°C for 30 s, and final extension for 5 min at 72°C. Step 2: Using NED, PET, VIC, or 6-FAM-m13-F primer and original R-primer and 1/50× concentration of PCR product from Step 1; PCR conditions were 94°C for 2 min, followed by 25 cycles at 94°C for 10 s, 52°C for 30 s and 72°C for 30 s. Final extension for 5 min at 72°C. PCR products were analyzed using fragment analysis at Yale’s DNA sequencing facility.

### Whole mount *in situ* hybridization (WMISH)

We obtained clones from Open Biosystems (Thermo Scientific). The DNA templates were *in vitro* transcribed with T7 High Yield RNA Synthesis Kit (E2040S) from New England Biolabs to synthesize digoxigenin-labeled antisense probes. Pronephric markers clones were obtained from Open Biosystems: *atp1a1*, IMAGE: 6988026; *slc7a8*, IMAGE: 53828775: *slc5a9*, IMAGE: 5308256; *smp-30*, IMAGE: 6999181; *hnf1-β,* TNeu141k07; *cdh16*, TNeu004i09. Whole mount *in situ* hybridization was performed according to the standard protocol with minor modifications [49,50]. We stained embryos using BM purple at room temperature or 4°C depending on each probe. If embryos needed to be genotyped after WMISH, fixation after staining was done with 4% paraformaldehyde instead of Bouin’s fixative to preserve DNA. After bleaching and equilibration in 100% glycerol, embryos were examined and photographed.

### *In vitro* fertilization and microinjections

*In vitro* fertilization and microinjection were done as previously described [51] and protocols available on our website (http://tropicalis.yale.edu). Embryos were injected either at one or at two cell stage with 0.5 or 1 ng of MO solution or at 2 cell stage with up to 500 pg *ccdc40* mRNA. *Ccdc40* mRNA was co-injected with 100 pg GFP mRNA. After injections, embryos were left in 1/9X MR + 3% Ficoll for 1 hr and then transferred to 1/9X MR supplemented with 50 μg/ml of gentamycin. Embryos were raised at 22-27°C until the appropriate stage for fixation or evaluating phenotype. *Pax8* translation blocking MO was obtained from Genetools, LLC. The sequence of the MO was: 5’ ATGCTGCTGTTGGGCATCTTCCTCC 3’. MO was co-injected with Alexa 488 dye (Invitrogen) as a lineage tracer.

### RT-PCR and cloning strategy

RNA was extracted from WT or from mutant embryos using Trizol solution according to the manufacturer's instructions (Invitrogen). A 1.5 μg aliquot of RNA was used to perform RT–PCR with SuperScript III Reverse Transcriptase (Invitrogen) using a gene specific primer located in the 3’UTR (UTR3-1R, 2R or 3R, Additional file 1: TableS 2) of *ccdc40* transcript according to the manufacturer’s instructions. PCR was performed using oligonucleotides in the 5’UTR or at beginning of the transcript (Additional file 1: Table S2). PCR was done with Phusion High-Fidelity DNA Polymerase (NEB) and conditions were as follows: 98°C for 2 min, followed by 39 cycles at 98°C for 15 s, 62°C for 30 s and 72°C for 3.5 min. Final extension was at 72°C for 5 min. Samples were then incubated with Taq polymerase for 30 min at 72°C to incorporate A overhangs for TOPO TA cloning (Invitrogen) and sequenced with several primers (Additional file 1: Table S2).

### Epidermal cilia-driven flow videos

A 6 cm petri dish was coated with 1% agarose. A small wedge shaped hole was cut into the agarose to position embryos with dorsal side up. 1 μL of 1/10× solution of 5 μm diameter polystyrene beads (Bangs Laboratories) were injected dorsally. Movies were captured using Canon EOS 5D Mark II DSLR camera.

## Abbreviations

SNP: Single nucleotide polymorphism; HTS: High throughput sequencing; WGS: Whole genome sequencing; BSA: Bulk segregant analysis; MUT: Mutant; WT: Wildtype; LOH: Loss of heterozygosity; RFLP: Restriction fragment length polymorphism; MO: morpholino; SEM: scanning electron microscopy; TEM: Transmission Electron Microscopy; SSLPs: simple sequence length polymorphisms.; WMSISH: Whole Mount *in situ* hybridization; ST: Stage.

## Competing interests

The authors declare that they have no competing interests.

## Authors' contributions

FDV, DB and MKK conceived and designed the experiments and wrote the manuscript. FDV, DB and MKK carried out the wet laboratory experiments. YK analyzed the HTS. MJG and MKK designed the exon capture array. FDV and DB contributed equally to this project. All the authors read and approved the final manuscript.

## Supplementary Material

Additional file 1 Additional file Figures 1–5 and Additional files Tables S1 and S2. Click here for file

Additional file 2 Movie 1: Epidermal cilia driven flow imaging of Wildtype embryos. Click here for file

Additional file 3 **Movie 2: Epidermal cilia driven flow imaging of *grinch *mutant.** Click here for file

Additional file 4 **Epidermal cilia driven flow imaging ofrescued *grinch *mutant embryo (left side injected).** Click here for file

Additional file 5 **Movie 4: Epidermal cilia driven flow imaging of rescued *grinch *mutant embryo (right side injected).** Click here for file
