# Supplementary material for: Exon capture and bulk segregant analysis: rapid discovery of causative mutations using high-throughput sequencing
Source: BMC Genomics. 2012 Nov 21;13:649. doi: 10.1186/1471-2164-13-649 (PMC3526394; doi:10.1186/1471-2164-13-649)
Supplement: Additional file 1 — Additional file Figures 1–5 and Additional files Tables S1 and S2. [file 1471-2164-13-649-S1.pdf]

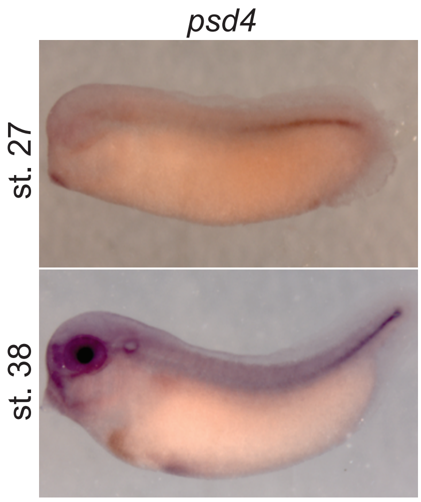

Supplementary Figure 1: *psd4* expression by WMISH at stages before pronephros are functional (st. 27) and when they are starting to function (st. 38).

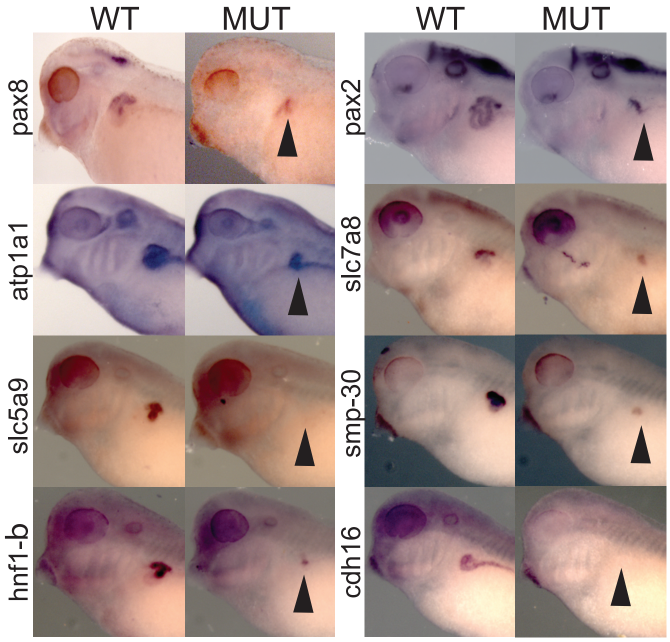

Supplementary Figure 2: Expression of pronephric markers in wt and ruby mutant embryos. Embryos from a heterozygous carrier cross were fixed at 36-37 stage and the expression of pronephric markers: *pax8* (*paired box 8*), *pax2* (*paired box 2*), *atp1a1* (*ATPase, Na+/K+ transporting, alpha 1 polypeptide*) , *slc7a8* (*solute carrier family 7 (amino acid transporter light chain, L system)*, *member 8*), *slc5a9* (*solute carrier family 5 (sodium/glucose cotransporter), member 9*), *smp-30* (*senescence marker protein-30*), *hnf1*-b (*HNF1 homeobox* b) and *cdh16* (*cadherin 16*), were studied by WMISH. Embryos were genotyped to confirm the mutation after imaging. Arrowheads indicate abnormal pronephros.

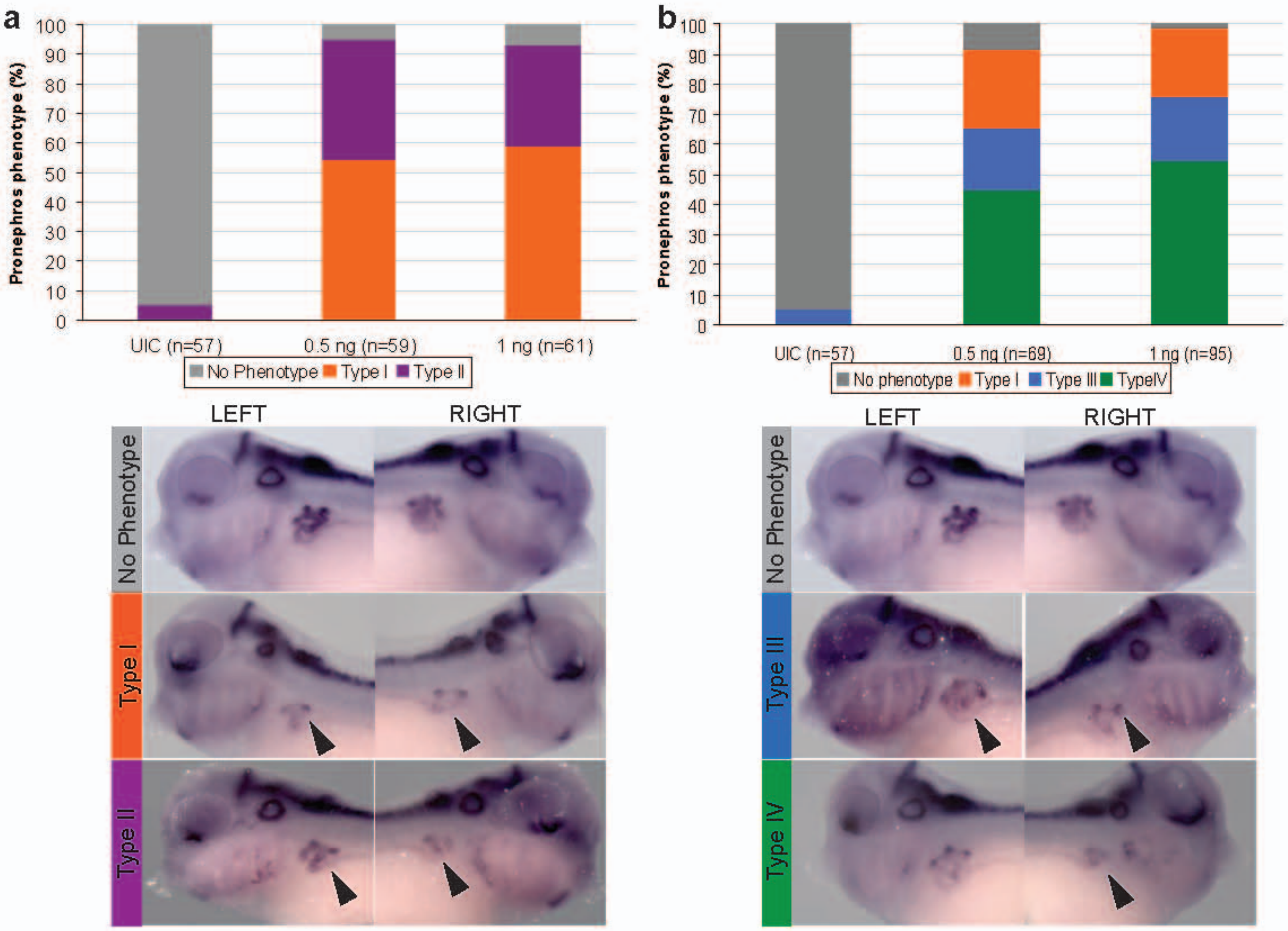

Supplementary Figure 3: Phenocopy of *ruby* mutant by *pax8* MO injection. *Pax8* morpholino was injected at one **(a)** or two cell stage (single cell injected) **(b)**. Embryos were fixed at st 36-37 and pronephric development was assessed by *pax2* expression. Abnormalities in pronephros development were classified: No phenotype; pronephros similar to uninjected controls (UIC); Type I: disrupted and abnormal pronephros similar to *ruby* mutants on both sides of the embryos. Type II: abnormal pronephros on one side and moderate abnormal pronephros on the other side, Type III: disrupted and abnormal pronephros on the injected side and moderate abnormal phenotype on the uninjected side. Type IV: disrupted and abnormal on the injected side and normal pronephros on the uninjected side. Images of *Pax2* WMISH showing each type of phenotype are shown. For Type III and IV, embryos shown were injected on the right side. Arrowheads indicate disrupted and abnormal pronephros.

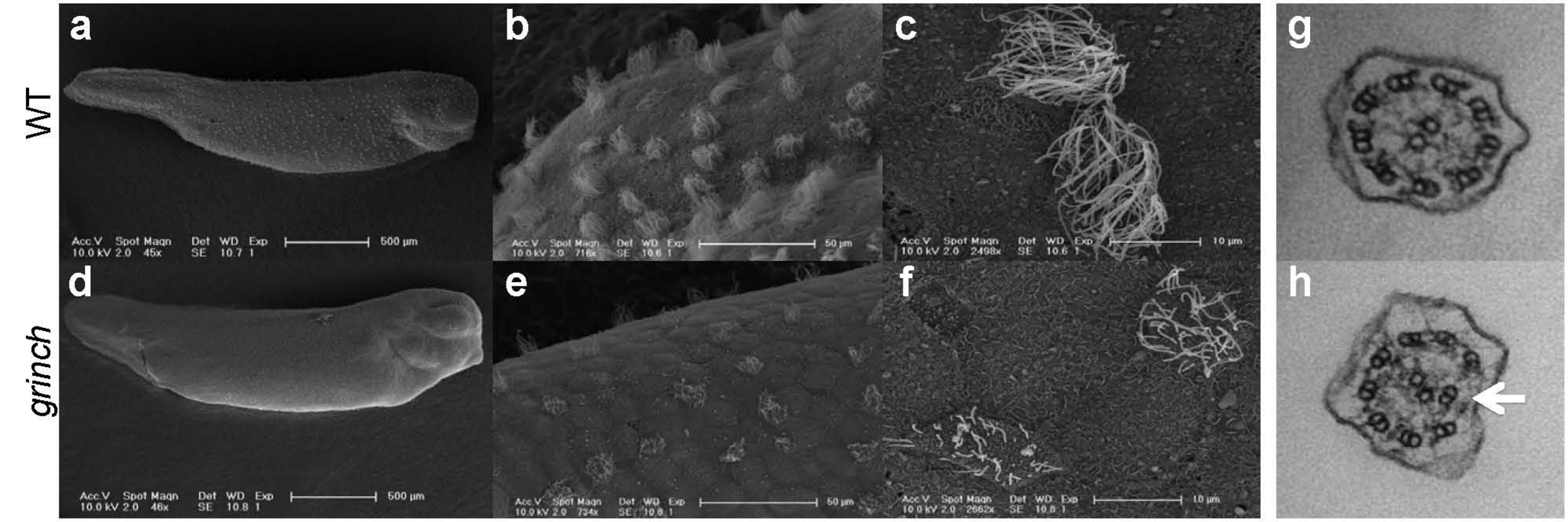

Supplementary Figure 4: SEM and TEM of WT and *grinch* embryos. **(a-f)** SEM; **(g,h)** TEM; **(a-c)** WT embryos; **(d-f)** *grinch* mutant embryos. **(f)** *grinch* mutants have fewer, shorter and abnormal cilia per cell. **(g)** WT cilia showing the 9+2 microtubule structure. **(h)** mutant cilia showing a displaced outer ring microtubule doublet (arrow). Embryos in **(a,d)** are lateral view with dorsal on top and anterior to the right.

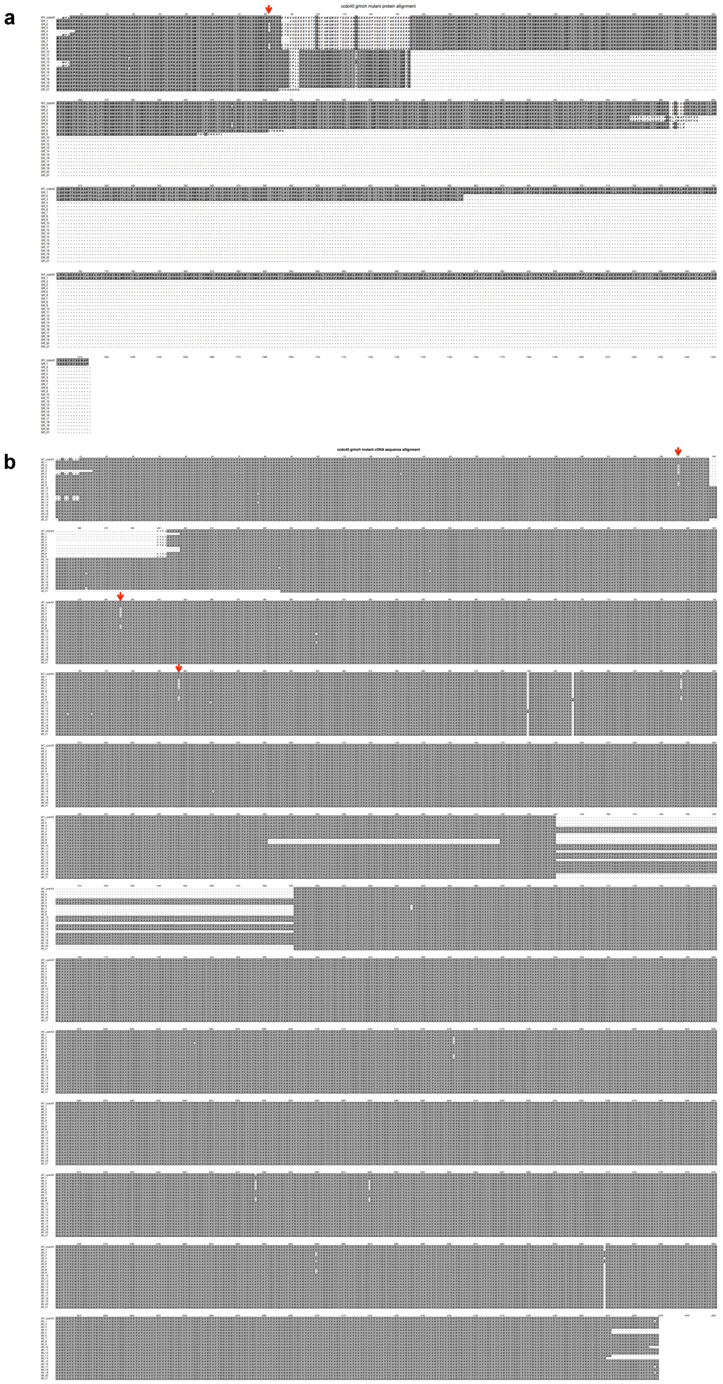

Supplementary Figure 5:
(a) Protein sequence alignment of the 21 *grinch* mutant clones. **(b)** cDNA sequence of the 21 mutant clones. Heterozygosity at the mutant locus (red arrows).

Supplementary Table 1: Genotyping primers used for fine mapping in *ruby* and *grinch*. n/a: not applicable.

Mutant	Marker	Genotyping Primers	Enzyme
ruby	1790	5' AATAAAGGTCCTTGCCTTGC 3'<br>5' CAATGTCAAGATGGCTTTGG 3'	StyI
	1211	5' CTGAGAGCCAGAATGAGAGG 3'<br>5' ATCATGCCTACACCAAGTGC 3'	PstI
	735	5' GCAGTCCTTCGACAATCAGC 3'<br>5' TTTGTATGGGCACTGGAACC 3'	PvuII
	1364	5' ATGAGGGAGAAATTCAGAGG 3'<br>5' GGTACTGGTGTGCGAATGG 3'	PstI
	1571	5' CTTCACCACAGTTCATCATGG 3'<br>5' CAGGAGACGCAAATAATCTTCC 3'	PstI
	1627	5' TCCGGTTCAATCTTCTCTGC 3'<br>5' CTCTAACGTTTCAGGGTTCC 3'	BamHI
	1789	5' GAGCCTTCTTAGTGGGATGG 3'<br>5' CAGTGCCATGAACAATCTCC 3'	PvuII
	1791	5' TGCTGATTCCCTGCACATAC 3'<br>5' GCAGCTCGAAGTGAAGATCC 3'	NspI
	1862	5' GGTGGCCACAAACGTTACC 3'<br>5' CGTTACCTCTGCAATCAAACC 3'	StyI
	2011	5' CCCATTCGAGAACTGGAAAA 3'<br>5' GGTTGTATTGGGAGCAATGG 3'	PsiI
	2043	5' CCACCCCTTTAAATGGAACC 3'<br>5' TTTGGCTGCCAGATACTCCT 3'	DraI
	2056	5' GCAAGCTGGGAAGGATGC 3'<br>5' GTTGGAAGTTGATGTTCTGC 3'	PstI
	2380	5' TGTGAAACAGGGAATGTAGG 3'<br>5' GGTAATGCCGAATGTCATGG 3'	PvuII
	6051	5' GCAAGCAGGAGCCTATAACG 3'<br>5' ATGACTGGCAAGCTGTTGG 3'	BamHI
grinch	008C01	5' AGACCCTGCCAAGAAAAGAC 3'<br>5' AGTCCACTCAGCCCTGTTC 3'	n/a
	016B06	5' AAAGGCAACAATAATGGCAG 3'<br>5' GCCAAGTCAGCATCTAATGG 3'	n/a
	100A10	5' ATTCTATCCCCTGATCCACC 3'<br>5' TGCAACTATGGGGCTTATTG 3'	n/a
	148E4	5' TCCCAGTCACGACGTATATTGAGGTGCCAGGATGC 3'<br>5' CACAAACAAGGCCGAAACTT 3'	n/a
	148M1	5' TCCCAGTCACGACGTGCATGGAACAAAACGTTGG 3'<br>5' ACCTTGGGTCAAAACATGGA 3'	n/a
	s148-783K	5' CATGTCATATTTGCCGTTGG 3'<br>5' AGCAACATTCAAGGGGTTGG 3'	SsiI
	s148-146K	5' CAGCAAGCTGGTTGTAGTGG 3'<br>5' GCCAGAGCTCTCAAGTAGAGG 3'	PstI
	s148-20K	5' CTTTTCAACAGCCTCTGATGG 3'<br>5' ATGCCAGAGTCCCATAAAGC 3'	AvaII
	s304-1.3M	5' TAGCTGCCCCTATCACCATC 3'<br>5' TACATGTCCCGGGCTTCTAT 3'	AlwNI
	s304-1M	5' CAGGGGTTGGACATGTAAGC 3'<br>5' AGGCAGATCCAGAGCTTTCC 3'	AvaII
	s304-875K	5' TCCCAGTCACGACGTCTCGAGCAATGAGTGACAGC 3'<br>5' TGGCACCAATAGGATTGACA 3'	n/a

	s304-219K	5' TCCCAGTCACGACGTAGAGCTGGCAGATGGCTAGA 3'<br>5' TGTGCCCATTTACCTGTTTG 3'	n/a
	560E1	5' TCCCAGTCACGACGTGGCATGGAGATGTTGCTCTT 3'<br>5' ATGGCCCATGACTCTCTGTT 3'	n/a
	560B2	5' TCCCAGTCACGACGTCATCCCAGTGCTTTGCTTTA 3'<br>5' GGCACTCACAAGGCTTTTATG 3'	n/a
	029C01	5' CGAGTTCGGCTATGACAGAC 3'<br>5' ATCCCTTTTGGGTTGGTTAC 3'	n/a
	045F05	5' TGCATTTTGTAATGGGGTG 3'<br>5' TTTGTCAGGCACAACAAGTC 3'	n/a
	N3_34	5' AGGAGATGCAATTTGGTTGG 3'<br>5' AGAGGCACACACAGGAAAGC 3'	n/a
	044G07	5' ACTGTGACCTTCCTACACGC 3'<br>5' ATTATCTTGTTGGGGACACG 3'	n/a
	006G10	5' TGCTTCAGGGGTCTTCATAC 3'<br>5' CGAGATAGCCAGATAGCCAG 3'	n/a

Supplementary Table 2: RT-PCR primers used for *grinch* transcript amplification and sequencing.

Name	Primer
UTR5-1F	5’ AGCGTCGCTTAGCAACAGTTAT 3’
UTR3-1R	5’ CCCAGCTACACAGCAGCTTATT 3’
UTR3-2R	5’ TCAATAGAGATTTACTAGCCAGTG 3’C
UTR3-3R	5’ CATTTCTTTTGTTTGTCAGTGTTTAG 3’
RT_full-F	5’ ATGTTCCTTAGCCCTAGATCTGG 3’
RT_full-R2	5’ TCAAGGTGCCCTCTCGGATGTA 3’
E2tE3-F	5’ AACAACCTCTTTCACCAAAAGC 3’
E3tE4-R	5’ ATAGGTCTTGAGAGCCGTCTGG 3’
E6tE7-F	5’ GAGAATATGAATCGGGATGTGC 3’
RT-3F	5’ TTCAGGAGGCTTTTAGTCTTGC 3’
RT-3R	5’ CCCTTTGCTTGGTTAGATTCAC 3’
RT-4F	5’ TACACTGGCAATTGAGAGGAAA 3’
RT-4R	5’ TCACTGACGGTTAACTGCTTCT 3’
RT-5F	5’ TCAGATCATGTTGTGGGAAAAG 3’
(Fl.)-m13-F	5’ TCCCAGTCACGACGT 3’
